# Assessment of Behavioral Predispositions of Selected Chicken Breeds for Use in Animal-Assisted Therapy: A Pilot Study

**DOI:** 10.3390/ani16101429

**Published:** 2026-05-07

**Authors:** Adam Szymański, Joanna Rosenbeger

**Affiliations:** Division of Poultry Breeding, Institute of Animal Breeding, Wroclaw University of Environmental and Life Sciences, 50-375 Wrocław, Poland; 130722@student.upwr.edu.pl

**Keywords:** animal-assisted therapy, chicken-assisted therapy, chicken breeds, behavior

## Abstract

The growth of interest in alternative forms of animal-assisted therapies (AAT) with chickens creates a need to identify breeds with appropriate behavioral predispositions. This pilot study evaluated the suitability of four breeds of ornamental chickens (*Gallus gallus domesticus*), Silkie bantam, Pekin bantam, Ko-Shamo, and Chabo daruma, for work in chicken-assisted therapy (CAT). Tests simulated an AAT session, assessing human approach and staying in their vicinity, reactions to sudden stimuli, and touch tolerance. A standard tonic immobility test was also performed. Significant interbreed differences were demonstrated (*p* < 0.05 for most tests). Pekin bantam showed the greatest therapeutic potential. Contrary to expectations, Silkie bantam obtained the weakest results, manifesting the highest fear levels and avoidance of contact. Ko-Shamo and Chabo daruma showed moderate predispositions. The tonic immobility test did not significantly differentiate the groups (*p* = 0.28), indicating it is not a sufficient alternative to the proposed behavioral tests. According to a seven-point predisposition scale for AAT, Pekin bantam was the breed best predisposed for AAT, while Silkie bantam achieved the worst results (*p* = 0.04). The results confirm that the breed is significant when choosing chickens for therapy and indicate the need for testing other chicken breeds for therapeutic work.

## Simple Summary

Animal-assisted therapy (AAT) involving chickens is gaining popularity; however, as with other species, not every breed possesses the necessary predispositions for this type of work. Ideal therapeutic animals should tolerate or seek human contact, accept physical touch, exhibit low reactivity to sudden stimuli, and display no aggression or fear in novel situations. This pilot study compared the reactions of four breeds—Silkie bantam, Pekin bantam, Ko-Shamo, and Chabo daruma—across a series of behavioral tests simulating a therapeutic session and various scenarios that may occur during its course. The results indicated that the Pekin bantam possesses the most suitable predisposition for AAT, as it most frequently remained in close proximity to humans, exhibited low reactivity to sudden stimuli, and demonstrated high tolerance to touch. Conversely, the Silkie bantam more frequently avoided close contact and performed poorly in tests evaluating human interaction. The Ko-Shamo and Chabo daruma yielded intermediate results, suggesting a requirement for more extensive adaptation and training prior to their use in therapy. These findings confirm that distinct behavioral predispositions for therapeutic work exist among chicken breeds.

## 1. Introduction

Animal-assisted therapy (AAT) is a method supporting the treatment and rehabilitation of people with disabilities, which consists of close contact with animals. Forms of therapeutic activities with the participation of an animal may consist of “animal-assisted activities” (AAA) that utilize companion animals [1] or “animal-assisted therapy” in a therapeutic context [2]. AAT has gained popularity, and the literature indicates numerous benefits resulting from this type of therapy [3]. It has been found that after AAT, 94% of elderly people experienced an improvement in mood, and in 88% of cases, anxiety levels decreased, participants felt more relaxed, and better socialization was observed [4]. It has been documented that AAT beneficially affects hemodynamic parameters in patients with heart failure and alleviates postoperative pain in children [5]. Research on the impact of patient interaction with cats has shown that contact with the animal leads to calming down—the patient’s cortisol decreases and the oxytocin level increases, which promotes stress reduction and mood improvement [6]. In a therapeutic context, felinotherapy also supports physical health—for example, in oncological patients, contact with a cat can accelerate the recovery process [7]. In animal-assisted therapy, the goal is not only the physical presence of the animal but also the interaction with it, which affects the emotional and social state of the therapy participant. In a four-month animal-assisted therapy program involving horses, teenagers reported a significant increase in perceived social support [8]. Therapies can be directed at various groups: children with developmental disorders, including the autism spectrum, and people with various disabilities, intellectual, hearing, visual, or physical. Therapies also find application in the cases of adults with anxiety disorders or depression, elderly people with mobility problems or loneliness, people in rehabilitation procedures, and socially maladjusted people with emotional or mental disorders. In recent years, alternative forms of AAT have enjoyed increasing interest, including not only dogs (canine-assisted therapy), cats (felinotherapy), or horses (hippotherapy), but also less typical animals that are part of non-standard forms of therapy, for example, therapies involving miniature rabbits (lagotherapy), alpacas (alpacatherapy), or dolphins (dolphin therapy). Alongside them, one of the developing forms of animal-assisted therapy is avian-assisted therapy, i.e., therapy with the participation of birds, within which we can distinguish chicken-assisted therapy (CAT), i.e., the use of chickens in the therapeutic process. It is believed that the presence of chickens can positively influence the mental well-being of patients, in particular children, the elderly, and people with disabilities. The history of CAT dates back to the 1700s when rabbits and chickens were used in England as an element of therapy for the mentally ill [9]. Although CAT is gaining popularity in the context of supporting mental and physical health, most research focuses on the participation of mammals—mainly dogs, cats, and horses. Therapies involving poultry are still poorly researched. Available studies on CAT are usually descriptive, popular science in nature, or based on case studies, without a systematic assessment of, for example, the predispositions of individual breeds for therapeutic work. There is a lack of studies that compare different chicken breeds in terms of their suitability for animal-assisted therapy. For example, in dogs, high excitability may increase the risk of injury to patients [10]. A calm temperament, the ability to adapt to various conditions, tolerance for contact with people and external stimuli, and a positive reaction to touch and handling are required [11]. The animal must also be healthy. Research shows that animals used in animal-assisted therapy should have a temperament that allows them to remain calm in various situations, and individuals should have a need to stay near humans. They should also not show tendencies toward aggressive behavior. All this is easier to achieve in the case of individuals showing innate predispositions. It is also pointed out that animal-assisted interventions can pose a threat to their welfare, especially when the selection of animals is inappropriate or the working conditions are not properly adapted [12]. Observations indicate that the voluntary participation of dogs in the role of co-therapists promoted the effectiveness of the therapy. In the study, most dogs manifested positive emotions, including joy, comfort, curiosity, and a willingness to play, which emphasizes the importance of their voluntary involvement in sessions [13].

Due to the growing interest in alternative forms of animal-assisted therapy, there is a need to identify the chicken breeds with the greatest therapeutic potential, which may contribute to improving the effectiveness of therapy, improving animal welfare in AAT, and increasing the safety of session participants. CAT can offer unique opportunities: close contact with a small animal, the possibility of observing its behavior, and interaction at a calm pace. The aim of this pilot study was to evaluate the suitability of four ornamental chicken breeds (the Silkie bantam, the Pekin bantam, the Ko-Shamo, and the Chabo daruma) for therapeutic work. For this purpose, the behavior of the birds was observed in various situations that simulated possible events during a therapeutic session and the course of the bird’s contact with a human itself. It was assumed that the breeds would differ from each other in terms of behavioral predispositions for animal-assisted therapy, and that the Silkie bantam and Pekin bantam would show more pro-social behaviors and lower intensity of fear reactions than the Ko-Shamo and Chabo daruma, which would make them more suitable for use in CAT. Thus, the research aimed to demonstrate which of the studied breeds possess innate predispositions for AAT.

## 2. Materials and Methods

### 2.1. Animals and Housing Conditions

All birds were hatched and reared at the Division of Poultry Breeding, Wrocław University of Environmental and Life Sciences. The animals were kept together in a pen measuring 2.20 × 2.70 m with access to an outdoor run measuring 2.40 × 10 m, which was made available during favorable weather conditions. At night, the birds were housed in the pen. The birds were fed commercial poultry feed adapted to their age. Access to feed and water was provided ad libitum. The birds were kept together from hatching under identical housing conditions to limit the impact of socialization and exclude early environmental factors that could affect the behavioral traits displayed by the birds later in life.

**Figure 1:**
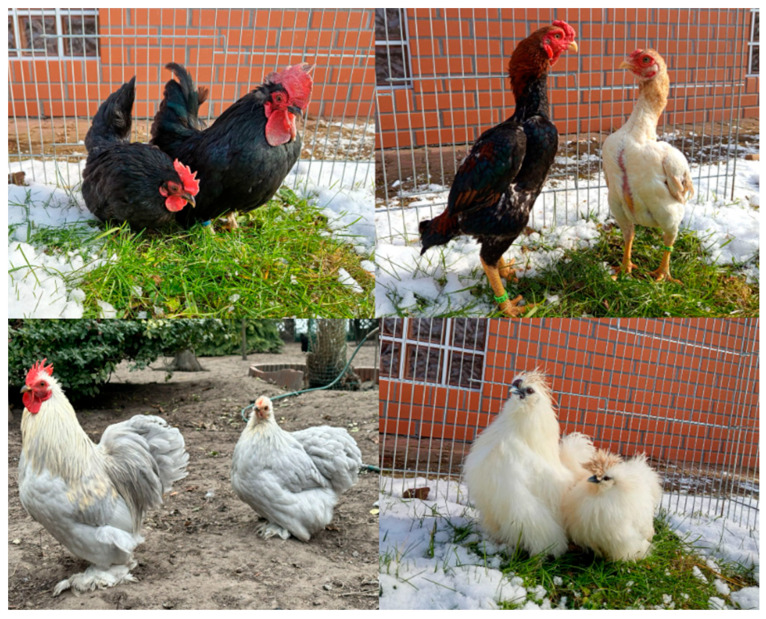
Appearance of the breeds used in the study. Each photograph shows adult birds of both sexes.

Four breeds of chicken were used in the experiment. Selection was based on diverse phenotypic characteristics, representing different genetic backgrounds and breeding purposes. This approach allowed for a broader evaluation of whether behavioral predispositions for therapeutic work are consistent across different morphotypes. These breeds were selected due to their small size and opinions on their reported potential suitability for therapeutic work: Silkie bantam—9 individuals (3 ♀ and 6 ♂), Pekin bantam—12 individuals (7 ♀ and 5 ♂), Ko-Shamo—9 individuals (6 ♀ and 3 ♂), and Chabo daruma—10 individuals (6 ♀ and 4 ♂). All birds were three months old. Differences in sex proportions in the groups resulted from the impossibility of predicting what the sex ratio of the hatched chicks would be. Similarly, the different sizes of the groups of individual breeds resulted from the impossibility of predicting the hatchability of the chicks. The Silkie bantam and Ko-Shamo breeds often have poor egg fertilization and hatchability results, so despite placing a significantly larger number of eggs for incubation than in the case of Chabo daruma and Pekin bantam, it was possible to obtain only 9 individuals of each of these breeds. Brief characteristics of the studied breeds are presented in Table 1, and their appearance is shown in Figure 1.

### 2.2. Behavioral Tests

In order to evaluate the behavioral predispositions of selected chicken breeds for CAT, seven behavioral tests were conducted. Due to the lack of standardized protocols for evaluating the therapeutic potential of chickens, these specific tests were developed by the authors for the purpose of this pilot study. All birds remained in optimal health throughout the testing period. The health status was monitored daily, and specific exclusion criteria were established: any birds showing signs of apathy, nasal or ocular discharge, or poor body condition (assessed by palpation of the pectoral muscles and keel bone) would have been excluded from the pilot study. Each test was carried out in the same test room measuring 2.20 × 2.70 m, during the same hours (9:00–15:00 h) and under unchanged environmental conditions (temperature, lighting). The applied tests were intended to determine the level of emotional reactivity, sensitivity to sudden environmental stimuli, and the degree of acceptance of touch and the presence of a human in conditions and situations designed to simulate situations that may occur during a therapeutic session. Before the start of the tests, the birds were habituated to human presence (through basic animal care in the form of providing feed, water, and cleaning). One day prior to testing, the chickens were habituated to the researcher’s presence and individually marked with colored animal sprays for later identification of the individual. To minimize observer bias and ensure consistent evaluation, all assessments were conducted in real time by a single observer. Measurements were carried out in the same order for all individuals. After each test, the chickens were returned to the rest of the flock for rest.

#### 2.2.1. Human Presence Test

The aim of the human presence (HP) test was to evaluate the behavior of the animal in the static presence of a human. The test was performed in two series of three repetitions each (on three consecutive days), with a 23-day break between series. Three zones were marked with chalk on the floor of the experimental room: Zone I < 0.5 m, Zone II 0.5–1 m, and Zone III > 1 m from the researcher. The researcher sat on a chair in the middle of Zone I. The time (s) spent by each bird in each zone was recorded.

#### 2.2.2. Approach People Test

The aim of the approach people (AP) test was to evaluate trust and readiness for contact with a human in a situation involving an interaction with a treat. At the beginning of the trial, each bird was placed in the corner of the room, at a distance of 1.5 m from the researcher, who sat with prepared treats in hand. Then, the researcher made attempts to call the bird by extending a hand with a treat and gradually tossing treats closer and closer to themselves. To confirm the desired response, a clicker was used to signal positive reinforcement each time the bird initiated movement toward the researcher. The test was carried out in two variants, each in three repetitions. In the first variant, lasting 5 min, it was evaluated whether the bird would approach the researcher at least once (approach: yes/no) and the latency to the first approach was noted. In the second variant, the observation time was shortened to 3 min, and the expected number of approaches was increased to three. The number of approaches and the latency to approach (s) were recorded.

#### 2.2.3. Touch Response Test

The aim of the touch response (TR) test was to evaluate the reaction to direct physical contact with the researcher. The test was performed over three consecutive days. During the test, the bird was held and touched by the researcher on the back, neck, and sides of the body. The duration of the touch was extended on subsequent days: day 1 (30 s), day 2 (45 s), and day 3 (60 s). Each day of the test, each of the wings was also lifted three times (six wing lifts in total). Reactions were defined as positive (acceptance of physical contact, no attempt to break away, calm reaction, no muscle tension, no signs of stress, no vocalization or quiet and gentle vocalizations, relaxed muscles during wing lifting) or negative (attempt to move away from the approaching hand, backing away, attempt to break away, tense body posture, trembling, loud vocalization, resistance during wing lifting, pecking at fingers, attempt to attack the hand).

#### 2.2.4. Sudden Acoustic Stimuli Test

The aim of the sudden acoustic stimuli (SAS) test was to evaluate sensitivity to unexpected acoustic stimuli. The test was conducted in three variants, each in three repetitions. In each variant, the bird was placed in the test room together with the researcher, who generated a sudden sound while the bird was busy foraging or exploring. The acoustic stimuli were, in order: 1—dropping seven plastic bowls onto the floor from a height of 1.5 m, 2—clapping hands loudly five times, and 3—coughing loudly 2–3 times. The bird’s baseline activity before the stimulus, the activity after the stimulus, based on which the actual reaction to the stimulus was determined (none, freeze, move, looking), and the time (s) of return to the previous activity were recorded, and the intensity of the reaction was categorized (0—no reaction, 1—weak reaction and lasting less than 30 s, 2—strong reaction, e.g., escape, jump and/or lasting more than 30 s).

#### 2.2.5. Unexpected Human Behavior Test

The aim of the unexpected human behavior (UHB) test was to evaluate tolerance to sudden, dynamic human behaviors. The test was performed in three variants with different stimuli (1—researcher jumping in place, 2—researcher waving arms for 5 s, 3—researcher throwing a marker toward the bird), and each of them in three trials. The bird’s baseline activity before the stimulus and the activity after the stimulus were noted, based on which the actual reaction to the stimulus was determined (none, freeze, move, looking, vocalization), as well as the duration of the reaction and the time of return to baseline activity (s).

#### 2.2.6. Chicken-Assisted Therapy Predisposition Test

Based on the tests described above, a protocol for evaluating predisposition to CAT was developed. In the test, a therapist supervised the interaction, while two “model patients” (individuals unknown to the birds) took seats in opposite corners of Zone I with bowls of food. The therapist remained in Zone III, recording the time, observing, and providing instructions regarding calling the birds and the movements and sounds made by the patients. Each test lasted 10 min. In the test, the chickens’ reaction to a new human, readiness for interaction with them, and the level of fearfulness in conditions similar to therapy were evaluated. Individuals could obtain a maximum of 7 points in the overall assessment: one point each for approaching a human (yes/no), staying near a human in Zone I (staying for more than half the time in Zone I from the patient), a positive reaction to touch (yes/no), and a total of 4 points for no reaction to four different situations in the form of unexpected stimuli (reaction to sudden, loud cough; hand clapping; human movement in the form of waving arms and legs; dropping a new object).

#### 2.2.7. Tonic Immobility Test

The aim of the tonic immobility (TI) test was to evaluate the level of fearfulness and susceptibility to stress. The test was repeated twice with a 12-day interval. Each bird was gently placed on its back and held for 3 s. If it stood up immediately, the trial was repeated a maximum of 3 times. The latency to the first head movement and to standing up (s) were recorded.

### 2.3. Statistics

The following statistical tests were used for the analyses: the Chi-square test, with an additional Fisher’s correction for small numbers of observations; Cramér’s V (in tests AP, TR, and SAS for intensity of reaction after stimulus action); and General Linear Model (GLM) analysis with Tukey’s post hoc test for level α = 0.05 (in tests HP for delay in return to previous activity, TI for reaction duration, and SAS and UHB for time of return to previous activity). Log-linear analysis of tables and counts was used in cases where data were nominal (in tests SAS and UHB for type of the behavior shown after stimulus). Due to small numbers of observations in some categories, similar categories of behaviors were merged in some cases (information about these cases is provided in Section 3). Due to the small size of the groups in the pilot study and the lack of meeting the assumptions of parametric tests, a non-parametric Kruskal–Wallis test with Dunn’s post hoc test was used to verify the hypotheses. A Benjamini–Hochberg (FDR) correction was also performed as appropriate for the pilot nature of the study. Analyses were performed in Statistica ver. 13.0 (TIBCO Software Inc., Palo Alto, CA, USA), while graphs were prepared in Minitab statistical software (ver. 17).

## 3. Results

### 3.1. Human Presence Test

Significant differences in the duration of stay near a human were demonstrated between breeds. In the first variant of the HP test, the Pekin bantam spent significantly the most time in Zone I (<0.5 m), while Ko-Shamo and Silkie bantam spent the least, and the duration of stay for the Chabo daruma was intermediate (*p* < 0.001; *F* = 6.66; *df* = 3). Neither the trial (*p* = 0.630; *F* = 0.46; *df* = 3) nor the interaction effect were statistically significant (*p* = 0.784; *F* = 0.53; *df* = 6). In Zone II (0.5–1 m), among all breeds, the Ko-Shamo stayed the longest (*p* = 0.001; *F* = 5.57; *df* = 3). Neither the trial (*p* = 0.150; *F* = 1.93; *df* = 2) nor the interaction effect was significant (*p* = 0.398; *F* = 1.05; *df* = 6). Consequently, the Silkie bantam and Chabo daruma spent the most time in Zone III (>1 m from the human) (*p* = 0.002; *F* = 5.36; *df* = 3), and neither the repetition (*p* = 0.925; *F* = 0.08; *df* = 2) nor the interaction effect influenced the birds’ duration of stay in this zone (*p* = 0.725; *F* = 0.61; *df* = 6). In the second variant of the HP test, the stay time in Zone I was again the longest for the Pekin bantam, followed by the Ko-Shamo, and was the shortest for the Silkie bantam and Chabo daruma (*p* = 0.001; *F* = 6.28; *df* = 3). The trial was not significant (*p* = 0.455; *F* = 0.79; *df* = 2), nor was the interaction effect (*p* = 0.998; *F* = 0.08; *df* = 6). In staying in Zone II, no differences were demonstrated between breeds (*p* = 0.080; *F* = 2.32; *df* = 3) or trials (*p* = 0.530; *F* = 0.64; *df* = 5), nor for the interaction effect (*p* = 0.998; *F* = 0.08; *df* = 6). In Zone III, the duration of stay was significantly longer for the Chabo daruma and Silkie bantam than for Pekin bantam (*p* = 0.001; *F* = 5.64; *df* = 3). No influence of the trial (*p* = 0.692; *F* = 0.37; *df* = 2) or the interaction effect (*p* = 0.988; *F* = 0.08; *df* = 6) was observed. The results are presented in Table 2.

### 3.2. Approach People Test

**Figure 2:**
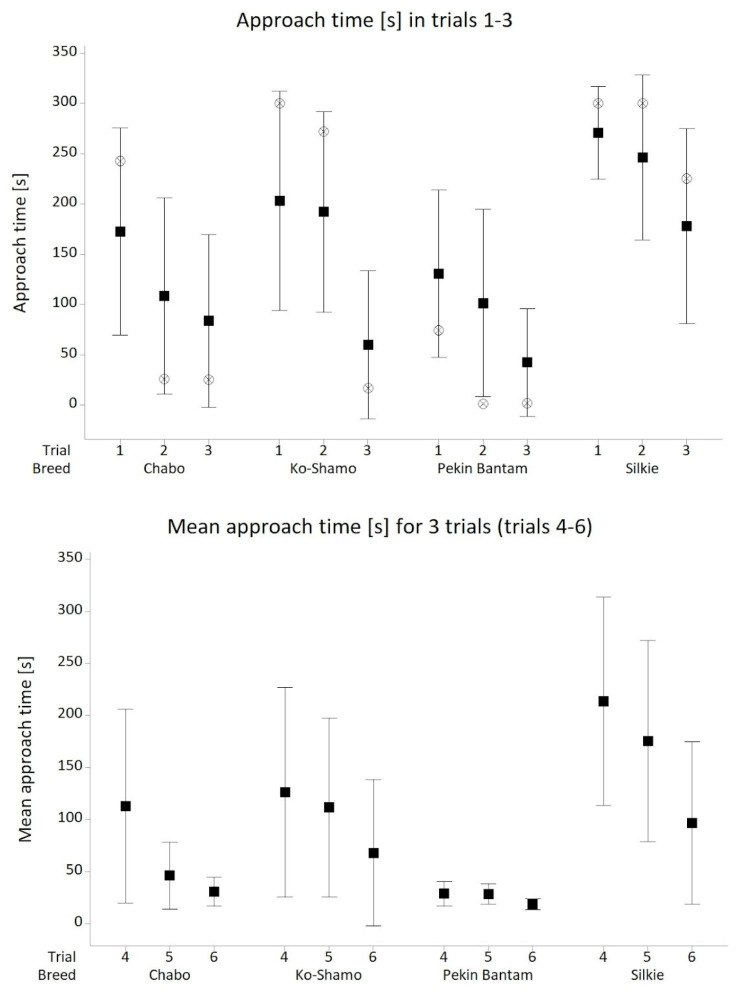
Interval plot for time of approach to human in 5 min trials (trials 1–3) and mean time of approach to human in 3 trials for 3 min (trials 4–6). Black squares show means; white circles show median.

**Figure 3:**
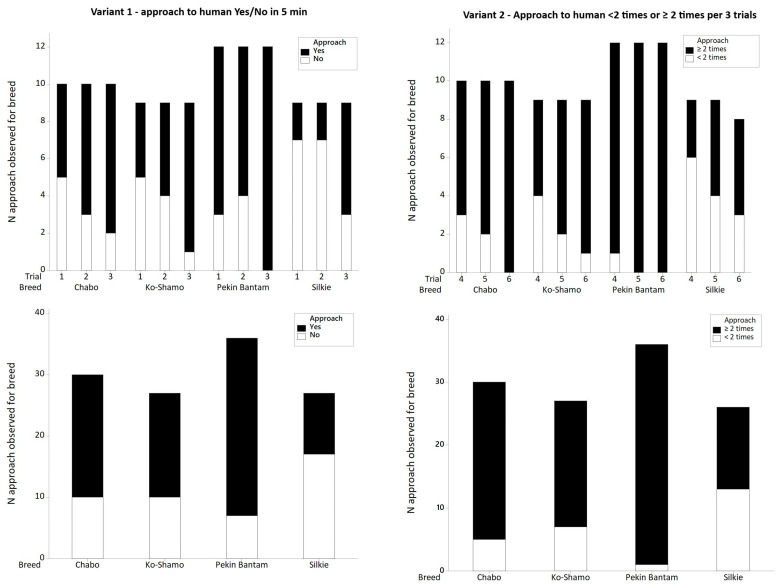
Variant 1—Approaches to human in 5 min trials (yes/no) for each trial (1–3) and summarized number of approaches to human, and Variant 2—Number of approaches to human in 3 min trials (<2 times, ≥2 times) for each trial (4–6) and summarized number of approaches.

In the first variant of the AP test examining the approach (yes/no) to a human (with a 5 min time limit), significant differences between breeds were demonstrated (*p* = 0.004, *df* = 3; χ^2^ = 13.23). The strength of the association was moderate (*V* = 0.33). The Silkie bantam approached least frequently (38.5%), the Pekin bantam most frequently (80.6%), and the Ko-Shamo and Chabo daruma at rates of 63.0% and 66.7% respectively (Figure 2). In the second variant of the test, where the number of times the bird approached the researcher was counted, the results were grouped into two categories: no approach or a single approach (fewer than < 2 times), and approached at least twice (approach ≥ 2 times). Again, a significant relationship between the frequency of approaches and breed was demonstrated (*p* < 0.001; *df* = 3; χ^2^ = 27.63). The results were confirmed using Fisher’s exact test due to small expected cell counts (*p* < 0.001). The strength of the association was high (*V* = 0.52). In 50% of cases the Silkie approached the researcher fewer than two times, while the Pekin bantam approached the researcher at least twice almost every time (97.2%). For the Ko-Shamo and Chabo daruma, these values were 73.1% and 86.7% respectively (Table 3). The distribution of the results achieved by individual breeds (including means and median) in subsequent trials is presented in Figure 2 and Figure 3.

### 3.3. Touch Response Test

Significant differences were demonstrated in the reaction of breeds to touch (*p* < 0.001, *df* = 3, χ^2^ = 51.86). The results were confirmed using Fisher’s exact test due to small expected cell counts (*p* < 0.001). The strength of the association was high (Cramér’s *V* = 0.66). The Silkie bantam reacted negatively to touch significantly more often (80%) than the other breeds, for which positive reactions constituted the majority: 80% for the Chabo daruma, 92.6% for the Ko-Shamo, and 94.4% for the Pekin bantam. In the reaction to wing lifting, statistically significant differences between breeds were demonstrated (*p* < 0.001, *df* = 3, χ^2^ = 19.30). The results were confirmed using Fisher’s exact test due to small cell counts (*p* < 0.001). The strength of the association was lower than during petting, but still moderate (Cramér’s *V* = 0.40). The Silkie bantam significantly more often showed a negative reaction (44%) than the other breeds. The Pekin bantam and Ko-Shamo showed mainly positive reactions (86.1% and 92.6%, respectively). In the case of the Chabo daruma, no negative reaction was observed. The distribution of responses to touch for individual breeds is presented in Table 4.

### 3.4. Sudden Acoustic Stimuli Test

**Figure 4:**
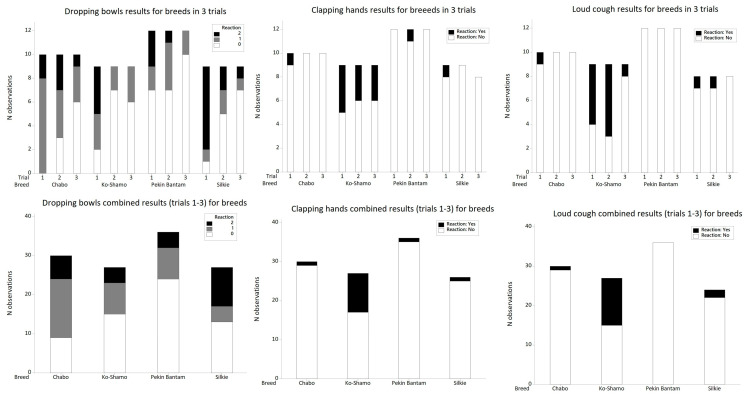
Intensity of breed reactions to sudden acoustic stimuli (SAS) test in subsequent trials and summarized number of reactions for all trials.

In the dropping bowls variant of the SAS test, a three-point scale for stimulus reaction was used. Statistical analyses showed a significant association between the intensity of the reaction and the breed (*p* = 0.009, *df* = 6; χ^2^ = 17.16) (Table 5). Results were confirmed using Fisher’s exact test due to small expected cell counts (*p* = 0.008). However, the strength of the association was low (Cramér’s *V* = 0.27). Compared to the other studied breeds, the Silkie bantam more frequently showed an intense reaction to the stimulus. An absence of reaction after the stimulus action was observed in the Pekin bantam, while the Chabo daruma and Ko-Shamo showed moderate reactions to the stimulus (Figure 4). After the stimulus action, no significant differences were observed between the breeds in the time of return to previous activity (*p* = 0.120; *F* = 1.99; *df* = 3), but this time was significantly longer in the first trial (*p* < 0.001; *F* = 16.71; *df* = 2). The interaction effect of breed and trial was significant (*p* = 0.002; *F* = 3.87; *df* = 6). The longest time of return to previous activity was observed in the case of Silkie bantam in the first trial, and the shortest in the Pekin bantam in the second and third trials, as well as in the Ko-Shamo in the second and third trials (Table 6).

In the clapping hands test variant, due to the small number of observations for reaction intensity categories 1 and 2, they were merged. Thus, two categories were distinguished: 0—no reaction to the stimulus, and 1—reaction to the stimulus present. Statistical analyses showed a significant association between the intensity of the reaction and the breed (*p* < 0.001, *df* = 3; χ^2^ = 22.12) (Table 5). Results were confirmed using Fisher’s exact test due to small expected cell counts (*p* < 0.001). However, the strength of the association was moderate (Cramér’s *V* = 0.43). Compared to the other breeds, in this test the Ko-Shamo more frequently showed a reaction to the stimulus. In turn, the Pekin bantam showed a tendency toward no reaction to the stimulus (Figure 4). The time of return to previous activity was significantly longer in the Ko-Shamo (*p* = 0.003; *F* = 4.83; *df* = 3). Neither the repetition (*p* = 0.319; *F* = 1.16; *df* = 2) nor the interaction effect was significant (*p* = 0.137; *F* = 1.66; *df* = 6) for the time of return to previous activity (Table 6).

In the loud cough variant, due to the small number of observations for reaction intensity categories 1 and 2, they were merged. Thus, two categories were distinguished: 0—no reaction to the stimulus, and 1—reaction to the stimulus present. Statistical analyses showed a significant association between the intensity of the reaction and the breed (*p* < 0.001, *df* = 3; χ^2^ = 30.35) (Table 5). Results were confirmed using Fisher’s exact test due to small expected cell counts (*p* < 0.001). However, the strength of the association was high (Cramér’s *V* = 0.51). As in the previous test, the Ko-Shamo reacted to the stimulus significantly more often, while the Pekin bantam did not show a reaction to the stimulus in any trial (Figure 4). The time of return to previous activity was significantly longer in the Ko-Shamo (*p* < 0.001; *F* = 9.89; *df* = 3), and it was also significantly longer in the first and second repetitions (*p* = 0.002; *F* = 6.60; *df* = 6). No significant influence of the interaction effect on the time of return to previous activity was observed (*p* = 0.125; *F* = 1.71; *df* = 6) (Table 6).

To examine the behavioral changes (freeze, looking, move or none) following the stimulus, log-linear analysis was employed. In most cases, the analysis revealed significant global interaction effects among the variables within the tested models (Table S1). The model built for time to return to previous activity in the dropping bowls variant showed the existence of a significant breed*reaction interaction effects for individual trials (*p* = 0.001) and trial*reaction for individual breeds (*p* = 0.031). Similarly in the case of the clapping hands variant, there was a significant breed*reaction interaction (*p* = 0.003), but not for trial*reaction (*p* = 0.151). The model for the loud cough variant was significant both in the breed*reaction (*p* < 0.001) and trial*reaction (*p* = 0.003) interaction. However, upon analysis of specific values, it was not demonstrated that any of the breeds significantly more often showed specific behaviors after the stimulus action (Tables S2 and S3). Only certain statistical tendencies were observed (a value between 1.000 and 1.959 or −1.959 and −1.000 was assumed) for the Pekin bantam, which in the loud cough trial 1 significantly more often showed freeze reactions, and in the dropping bowls trial 2 showed a tendency for a more frequent freeze reaction. The Ko-Shamo, in turn, showed certain tendencies toward no reaction (test variant dropping bowls, trial 2; test variant clapping hands, trial 1).

### 3.5. Unexpected Human Behavior Test

In the first variant of the UHB test (jump), no differences were observed between breeds in the time of return to previous behavior after the stimulus action (*p* = 0.727; *F* = 0.44; *df* = 3), similarly for subsequent trials (*p* = 0.067; *F* = 2.78; *df* = 2), the interaction effect was also not significant (*p* = 0.633; *F* = 0.72; *df* = 6). In the second variant (arm swing), the results also turned out to be non-significant for breed (*p* = 0.684; *F* = 0.50; *df* = 3), trial (*p* = 0.612; *F* = 0.49; *df* = 2), and the interaction effect (*p* = 0.526; *F* = 0.86; *df* = 6). However, the tests showed differences in the case of the third variant (throw marker). The longest time of return to previous activity after the stimulus action was shown by the Ko-Shamo, followed by the Silkie bantam and Pekin bantam, and the Chabo daruma exhibited the shortest recovery period (*p* = 0.049; *F* = 2.70; *df* = 3). The reaction was significantly longer in the first trial than in subsequent ones (*p* = 0.004; *F* = 5.88; *df* = 2). The interaction effect was not statistically significant (*p* = 0.412; *F* = 1.03; *df* = 6).

To examine the behavioral changes (into Freeze, Looking, Move, Vocalization or None) following the stimulus, log-linear analysis was employed. In the analysis for jump variant, the model was significant for breed*reaction (*p* = 0.006) and for trial*reaction (*p* < 0.001). The model in the variant throw marker test was non-significant for both breed*reaction (*p* < 0.236) and trial*reaction (*p* = 0.647). Similarly in the variant arm swing for breed*reaction (*p* < 0.381) and trial*reaction (*p* = 0.396). The values for the model are presented in Table S1. An analysis of specific values did not demonstrate that any of the breeds showed specific behaviors significantly more frequently after the stimulus action (Tables S2 and S3). Only certain statistical tendencies were observed (standardized residuals between 1.000 and 1.959 or −1.959 and −1.000). The Pekin bantam showed a greater tendency toward freezing in the variants jump (trial 2) and arm swing (trial 1), and only once showed a lower frequency of the freezing reaction (arm swing trial 3). In turn, the Ko-Shamo more frequently showed tendencies toward no reaction (variant jump, trial 2).

### 3.6. Chicken-Assisted Therapy Predisposition Test

Significant differences were demonstrated between breeds in the number of obtained points of predisposition for CAT (*p* = 0.040; *df* = 3; *H* = 8.30). Pekin bantam achieved the best results: on average 5.5 points (*z* = 2.77), then the Chabo daruma (*z* = −0.66) with a score of 4.0, the Silkie bantam (*z* = −1.07) with a score of 3.9, and the Ko-Shamo (*z* = −1.30) with a score of 3.9. Thus, the Pekin bantam significantly differed from the other breeds; however, after applying the Benjamini–Hochberg (FDR) correction, these differences were reduced to statistical tendencies (Table 7).

### 3.7. Tonic Immobility Test

No significant differences were demonstrated between breeds (*p* = 0.283; *F* = 1.32; *df* = 3) or trials (*p* = 0.069; *F* = 2.90; *df* = 2) in the duration of tonic immobility measured until the moment of head movement. In the case of the same test considering the time until the bird stands up, no significant differences were demonstrated between breeds (*p* = 0.387; *F* = 1.04; *df* = 3), but the trial was significant (*p* = 0.026; *F* = 4.07; *df* = 2). In the third repetition, the birds stood up faster than in the first one.

## 4. Discussion

Animals used in therapeutic work should be characterized by an appropriate temperament, a low level of reactivity to environmental stimuli, and ability to adapt to new situations [15]. It should be noted that these birds, due to their unique appearance, behaviors, and usually small body sizes, may constitute an interesting alternative to traditionally used species of therapeutic animals. In many therapeutic contexts, chickens could effectively complement or even offer a viable alternative to the most common species, such as equines, canines, or dolphins, particularly where space and logistics are limited. Differences in the size, conformation, coloration, and behavior of animals can influence their perception by the patient and readiness for interaction. Similarly, in poultry, there is great breed variability, which creates the possibility of adapting the animals to the needs of therapy participants. However, the basic condition for safe animal-assisted therapy, both for the animal and the patient, is an appropriate evaluation of behavioral reactions to stimuli. Research results suggest that breed differences play a key role in shaping behavioral predispositions in the case of dogs, especially in the context of their functional suitability [16]. Due to their predispositions, the breeds most frequently used in therapeutic work are the Labrador Retriever and Golden Retriever. This results from their low level of aggression and high trainability [10]. Thus, selecting the appropriate breed is the initial stage in identifying individuals with specific predispositions. Underlying these differences are largely physiological factors, such as the stress response, which in turn have a genetic basis, and can therefore be improved through selective breeding. Research indicates that stress responses can vary significantly between breeds, and even between lines within a breed [17]. Birds may exhibit different levels of emotional reactivity, and even preferences regarding environmental enrichment and the type of feed [18]. These differences are evident from the very first day of life, and although certain reactions, such as fearfulness, may change with age, a clear tendency towards breed-specific behaviors is evident from the outset [19]. It has been shown that different lines of chickens, such as White Leghorn and their wild ancestor Red Junglefowl, differ in terms of both behavioral and physiological responses to stress [20]. These differences result from selection and indicate that fear reactions and stimulus response patterns are heritable [20,21]. Consequently, the varied reactions observed in the chicken breeds examined in the present pilot study most likely stemmed not from environmental factors or the animals’ previous experiences (as all individuals had identical exposure to humans, were raised in the same conditions, and were of the same age), but from genetic determinants.

In dogs, as the animals most frequently used in AAT, individual assessments of predispositions for therapeutic work are standardly performed [10,22]. Tools such as the Positive and Negative Affect Scale PANAS [23], C-BARQ questionnaires [24], or Monash Canine Personality [25] are most commonly used. Given the growing interest in alternative species for animal-assisted therapy, developing similar guidelines for other animals, including chickens, would be highly beneficial. This would indirectly facilitate identifying the predispositions of individual breeds for therapeutic work and establishing an assessment scale for the studied species. While developing a definitive assessment scale for chickens was beyond the scope of this pilot study, this research is a pioneer in the preliminary standardization of parameters to evaluate potential candidates for CAT. Our findings provide a preliminary foundation for identifying behaviors that could be incorporated into such an assessment.

The obtained results demonstrated that the Pekin bantam breed exhibits the greatest predisposition for therapeutic work across nearly all tests, owing to its tendency to stay near humans, low reactivity to sudden stimuli, rapid recovery to a state of calmness, and high tolerance to touch. Contrary to expectations, the Silkie bantam, a breed considered in popular opinion to be good for AAT, achieved the worst results in many tests. Specifically, Silkie bantams performed worst in tests evaluating approaching and staying near a human. These results suggest heightened caution and fearfulness, which may limit the suitability of this breed for activities requiring close human interaction. To maintain animal welfare during a therapeutic session, it is essential that the animal enters into interactions willingly. Additionally, Silkie bantams showed the lowest tolerance for touch and body manipulation. Given that physical contact is a fundamental aspect of AAT and plays a key role in the benefits of human–animal interaction, it is desirable for therapeutic animals to exhibit positive reactions while being touched [26]. However, the possibility cannot be excluded that these results are related specifically to the choice of Silkie bantams rather than the large Silkie variety. Although the birds selected for this pilot study are considered a variety within the breed [14], it is possible that this influenced the findings, which appear to contradict standard breed descriptions [27]. Nevertheless, there is no existing literature comparing the temperament of these two Silkie forms, and information is currently limited to breeder opinions. Consequently, the observations in the present pilot study refer specifically to the miniature version, and caution should be exercised when generalizing these findings to the entire breed. The Silkie bantams also exhibited the strongest and most prolonged reactions to sudden stimuli. Given the multitude of novel stimuli that may occur during a therapeutic session, such reactivity exposes the animal to stress, potentially triggering escape or avoidance behaviors. Research has shown that therapeutic dogs, in response to work-induced stress, can display a range of reactions, from avoidance and freezing to aggression [28]. During a therapeutic session, the animal’s signals must be analyzed continuously by the therapist to prevent dangerous situations. In mammals, many aspects of body language and facial expressions are similar across species (e.g., baring teeth, tucking the tail), allowing humans to interpret them correctly; this is not the case with birds. Unlike mammals, birds, including chickens, lack complex facial expressions, and their facial mimicry is limited to “facial behaviors” such as blinking [29]. Bird communication is primarily based on body posture, feather ruffling, or changes in the color intensity of bare skin elements [30]. This may make it difficult for a patient unfamiliar with avian behavior—or an inexperienced therapist—to accurately assess the animal’s emotional state. Therefore, the selection of appropriate chicken breeds for AAT is of paramount importance.

In the context of therapeutic applications, low fear reactivity and emotional stability are crucial, as they ensure calm and predictable animal behavior. The findings suggest that the Ko-Shamo exhibits greater reactivity to sudden acoustic stimuli, which may limit its suitability for AAT, particularly in environments with unpredictable external factors. In most trials, the Ko-Shamo and Chabo daruma demonstrated moderate predispositions for therapeutic work; however, they showed high tolerance to touch, comparable to the Pekin bantam, and even superior results during wing manipulation. It is noteworthy that the Ko-Shamo is a gamefowl breed described as having a lively temperament; while they are trusting and easily tamed by humans, they remain combative toward conspecifics [31]. This breed was selected for testing as a potential AAT candidate alongside the Pekin bantam and Silkie bantam, which are traditionally regarded as predisposed for CAT. The Chabo daruma, conversely, is considered a docile breed with no predisposition toward aggression. Thus, beyond their small body size, these breeds share a reputation for being balanced and non-aggressive toward humans.

However, the primary focus of poultry selection historically remains production traits (egg-laying or body weight) or, in ornamental breeds, specific morphological characteristics. Selection for behavioral traits, although practiced [32], usually remains secondary to productivity and conformation. Therefore, targeted selection for desired behavioral traits should be considered alongside production goals. The studied breeds exhibit relatively low egg production (average annual egg count: Chabo 30–40; Ko-Shamo and Pekin bantam 80; Silkie 120) [14] compared to commercial strains. While this may reduce selection efficiency relative to high-production breeds, the high number of offspring and rapid sexual maturation in poultry allow for faster fixation of desired behavioral traits compared to other therapeutic animal species.

A study by Bryan [33] demonstrated that frequent eye contact with humans was associated with lower fear reactivity in birds compared to individuals with infrequent caregiver interaction. Chickens are capable of discriminating between various human postures and exhibit heightened responses to behaviors perceived as potentially threatening, such as direct eye contact or raised hands [34]. Our results revealed a decrease in reaction intensity across subsequent trials, indicating a habituation process that is critical for determining therapeutic suitability. This suggests that with an adequate habituation period, all studied breeds might potentially perform effectively in AAT, although certain breeds would require significantly more extensive training.

Conducting the above tests in various variants is time-consuming, so we decided to check whether the tonic immobility test can indicate the birds’ predisposition to AAT. Since it is easy to perform and serves to evaluate the fear level in poultry (longer time remaining in immobility is interpreted as higher fear reactivity of the animal), it could constitute a simple and quick alternative [35,36]. Contrary to expectations, no differences between breeds were found, even though a series of tests, including the pilot study, showed that Pekin bantam is the best breed for CAT. Therefore, these findings suggest that the TI test does not provide a reliable basis for assessing suitability for AAT in the studied breeds.

The obtained results indicate that breed affiliation is a factor determining suitability for AAT. Observed behavioral differences confirm that predispositions for therapeutic work are contingent upon specific temperament traits and reactivity to environmental stimuli. Notably, popular assumptions regarding a docile temperament are not consistently supported by empirical observation, as evidenced by the performance of the Silkie bantam in this study. According to the tests, the best predisposed breed turned out to be the Pekin bantam, demonstrating the capacity for demanding therapeutic tasks that require low reactivity to stimuli, high sociability, and a strong tolerance for physical manipulation. Other breeds (such as the Chabo daruma and Ko-Shamo) may be better suited for therapeutic contexts characterized by lower stimulus intensity or more controlled environments. The performance of these two breeds reflects a high level of potential, suggesting that with a more focused and extended socialization process, they can fully adapt to their role in CAT.

Future research should focus on the development of standardized protocols for evaluating the suitability of chickens for AAT. Such protocols should incorporate both reactivity tests and the assessment of pro-social behaviors and adaptive capacities to enhance the efficacy and safety of therapeutic interventions involving poultry.

## 5. Conclusions

Our results indicate that chicken breed constitutes an important factor determining suitability for CAT. The assessment of behavioral predispositions of selected breeds for work with humans allowed for the observation of differences between the analyzed breeds. Both individual tests and the seven-point scale of predisposition for AAT developed on their basis showed that the Pekin bantam possesses the greatest potential for therapeutic work (staying near a human, tolerance to touch, low reactivity to unexpected acoustic stimuli and human behaviors), while the Silkie bantam turned out to be the worst predisposed due to the tendency to avoid staying near a human or lower tolerance to touch. The Ko-Shamo and Chabo daruma presented intermediate results, while progressive adaptation in subsequent trials was observed in them. Both of these breeds showed good tolerance to touch, despite greater reactivity to sudden stimuli, which may constitute a potential obstacle in using them in AAT. Furthermore, our results show that the tonic immobility test is not an effective predictor of the suitability of chicken breeds for AAT. This emphasizes the necessity for further research and refinement of suitability tests for chickens for CAT, which are based on various aspects of predisposition, rather than a simple assessment of fearfulness.

The results emphasize the great importance of appropriate selection of chicken breeds for therapeutic purposes to improve the effectiveness of CAT itself, as well as the welfare of the involved animals. Individuals with low fearfulness, low reactivity, and high tolerance of contact with humans are the most desirable. Future research should also test the predispositions of other breeds for therapeutic work and develop a protocol for assessing suitability for CAT.

## Figures and Tables

**Table 1:** Characteristics of the chicken breeds studied according to Roszkowski et al. [14].

Breed	Body Mass	Breed Description
Silkie bantam	♂ 600 g ♀ 500 g	Angular silhouette. Feathers lack hooks, the skin is black in all color varieties, five-toed feet. Feathers form a crest on the head. Lack the ability to fly.
Pekin bantam	♂ 850 g ♀ 750 g	Spherical silhouette, soft profuse plumage. Wide back, short legs, feathered shanks.
Ko-Shamo	♂ 800–1000 g ♀ 600–800 g	Upright posture, short, close-fitting plumage to the body, lack of feathers on the edge of the sternum. Walnut-shaped comb and wattles small.
Chabo daruma	♂ 780–850 g ♀ 580–670 g	Low silhouette, short legs, vertically carried tail. Large comb and wattles.

**Table 2:** Multiple comparisons of mean results of time spent in different zones for breeds, trials, and breed*trial (Tukey’s post hoc test). Means marked with different letters of the alphabet differ statistically significantly at the significance level α = 0.05 (by columns).

	Test Variant 1			Test Variant 2		
	Zone I (<0.5 m)	Zone II (>0.5–1 m)	Zone III (>1.0 m)	Zone I (<0.5 m)	Zone II (>0.5–1 m)	Zone III (>1.0 m)
Silkie bantam	71.78 ^b^	22.11 ^b^	86.11 ^a^	124.33 ^b^	7.21 ^a^	47.63 ^a^
Pekin bantam	141.86 ^a^	16.92 ^b^	21.47 ^b^	171.86 ^a^	5.86 ^a^	2.28 ^b^
Ko-Shamo	74.22 ^b^	48.67 ^a^	57.70 ^ab^	142.11 ^ab^	17.11 ^a^	20.78 ^ab^
Chabo daruma	100.17 ^ab^	17.13 ^b^	64.03 ^a^	108.57 ^b^	23.43 ^a^	48.00 ^a^
Trial 1	88.06 ^a^	34.70 ^a^	57.70 ^a^	126.60 ^a^	17.82 ^a^	35.580 ^a^
Trial 2	102.03 ^a^	23.73 ^a^	54.25 ^a^	140.10 ^a^	12.03 ^a^	27.25 ^a^
Trial 3	100.93 ^a^	20.19 ^a^	60.04 ^a^	143.46 ^a^	10.36 ^a^	26.18 ^a^
Silkie bantam*trial 1	71.56 ^a^	43.44 ^ab^	65.00 ^a^	111.75 ^a^	13.75 ^a^	54.50 ^a^
Silkie bantam*trial 2	76.00 ^a^	12.56 ^ab^	91.44 ^a^	123.38 ^a^	6.38 ^a^	47.75 ^a^
Silkie bantam*trial 3	67.78 ^a^	10.33 ^ab^	101.89 ^a^	137.88 ^a^	1.50 ^a^	40.63 ^a^
Pekin bantam*trial 1	153.33 ^a^	12.00 ^b^	15.42 ^a^	164.92 ^a^	12.50 ^a^	2.58 ^a^
Pekin bantam*trial 2	140.58 ^a^	21.67 ^ab^	17.75 ^a^	172.67 ^a^	3.08 ^a^	4.25 ^a^
Pekin bantam*trial 3	131.67 ^a^	17.08 ^ab^	31.25 ^a^	178.00 ^a^	2.00 ^a^	0.00 ^a^
Ko-Shamo*trial 1	53.67 ^a^	63.67 ^a^	63.78 ^a^	136.33 ^a^	17.33 ^a^	26.33 ^a^
Ko-Shamo*trial 2	78.22 ^a^	47.78 ^ab^	54.00 ^a^	145.56 ^a^	17.56 ^a^	16.89 ^a^
Ko-Shamo*trial 3	90.78 ^a^	34.56 ^ab^	55.33 ^a^	144.44 ^a^	16.44 ^a^	19.11 ^a^
Chabo daruma*trial 1	73.70 ^a^	19.70 ^ab^	86.60 ^a^	93.40 ^a^	27.70 ^a^	58.90 ^a^
Chabo daruma*trial 2	113.30 ^a^	12.90 ^ab^	53.80 ^a^	118.80 ^a^	21.10 ^a^	40.10 ^a^
Chabo daruma*trial 3	113.50 ^a^	18.80 ^ab^	51.70 ^a^	113.50 ^a^	21.50 ^a^	45.00 ^a^

**Table 3:** Number of observations, frequency of observations (%) and adjusted residuals for approach people (AP) test in both variants. An asterisk (*) denotes values significantly deviating from the norm (above 1.96 or below −1.96).

Breed	Variant 1—Approach Human Yes/No in 5 min		*n*
Silkie bantam	10 (38.5%); −2.67 *	16 (61.5%); 2.67 *	26
Pekin bantam	29 (80.6%); 2.27 *	7 (19.4%); −2.27 *	36
Ko-Shamo	17 (63.0%); −0.01	10 (37.0%); 0.01	27
Chabo daruma	20 (66.7%); 0.66	10 (33.3%); −0.66	30
**Variant 2—Approach Human < 2 Times or ≥2 Times per 3 Trials**			
Silkie bantam	13 (50.0%); 3.47 *	13 (50.0%); −3.47 *	26
Pekin bantam	1 (2.8%); −4.48 *	35 (97.2%); 4.48 *	36
Ko-Shamo	7 (26.9%); −0.61	19 (73.1%); 0.61	27
Chabo daruma	4 (13.3%); −2.06 *	26 (86.7%); 2.06 *	30

**Table 4:** Frequency of positive and negative responses of the studied breeds in reaction to touch in the touch response (TR) test. The following are provided: the number of observations, percentage of reactions, and adjusted standardized residuals. An asterisk (*) denotes values significantly deviating from the norm (above 1.96 or below −1.96).

Breed	Reaction Positive	Reaction Negative	*n*
**Petting**			
Silkie bantam	5 (20.0%); −4.31 *	20 (80.0%); 4.31 *	25
Pekin bantam	34 (94.4%); 4.62 *	2 (5.6%); −4.62 *	36
Ko-Shamo	25 (92.6%); 3.72 *	2 (7.4%); −3.72 *	27
Chabo daruma	24 (80.0%); 1.31	6 (20.0%); −1.31	30
**Wing lifting**			
Silkie bantam	15 (55.6%); −2.62 *	12 (44.4%) 2.61 *	27
Pekin bantam	31 (86.1%); 1.44	5 (13.9%); −1.44	36
Ko-Shamo	25 (92.6%); 1.97 *	2 (7.4%); −1.97 *	27
Chabo daruma	30 (100.0%); 2.69 *	0 (0.0%); −2.69 *	30

**Table 5:** Frequency of occurrence of reaction intensity (0–2) in response to sudden acoustic stimuli (SAS) in three test variants. The following are given in order: number of observations, percentage of reactions, and adjusted standardized residuals. An asterisk (*) denotes values significantly deviating from the norm (above 1.96 or below −1.96).

Dropping Bowls				
**Breed**	**Reaction 0 *n* and %**	**Reaction 1 *n* and %**	**Reaction 2 *n* and %**	***n***
Silkie bantam	13 (48.15%); −0.32	4 (14.81%); −1.86	10 (37.04%); 2.51 *	28
Pekin bantam	24 (66.67%); 2.27 *	8 (22.22%); −1.10	4 (11.11%); −1.59	36
Ko-Shamo	15 (55.56%); 0.56	8 (29.63%); 0.06	4 (14.81%); −0.77	27
Chabo daruma	9 (30.00%); −2.64 *	15 (50.00%); 2.90 *	6 (20.00%); 0.00	30
**Clapping Hands**				
**Breed**	**Reaction 0 *n* and %**	**Reaction 1 *n* and %**	***n***	
Silkie bantam	25 (92.59%); 0.78	2 (7.41%) −0.78	27	
Pekin bantam	35 (97.22%); 1.99 *	1 (2.78%); −1.99 *	36	
Ko-Shamo	17 (62.96%); −4.66 *	10 (37.04%); 4.66 *	27	
Chabo daruma	29 (96.67%); 1.64	1 (3.33%); −1.64	30	
**Loud Cough**				
**Breed**	**Reaction 0 *n* and %**	**Reaction 1 *n* and %**	***n***	
Silkie bantam	22 (88.00%); 0.25	3 (12.00%); −0.25	25	
Pekin bantam	36 (100.00%); 2.85 *	0 (0.00%); −2.85 *	36	
Ko-Shamo	15 (55.56%); −5.34 *	12 (44.44%); 5.34 *	27	
Chabo daruma	29 (96.67%); 1.89	1 (3.33%); −1.89	30	

**Table 6:** Multiple comparisons (GLM) of mean results of time to return to previous activity in response to variants of sudden acoustic stimuli (SAS) test for breeds, trials, and breed*trial (Tukey’s post hoc test). Means marked with different letters of the alphabet differ statistically significantly at the significance level α = 0.05.

Test	Dropping Bowls	Clapping Hands	Loud Cough
Silkie bantam	39.85 ^a^	2.15 ^a^	3.00 ^b^
Pekin bantam	14.33 ^a^	1.97 ^b^	2.25 ^b^
Ko-Shamo	25.85 ^a^	12.41 ^b^	8.81 ^a^
Chabo daruma	28.03 ^a^	1.70 ^b^	2.63 ^b^
Trial 1	57.23 ^a^	4.09 ^a^	6.07 ^a^
Trial 2	18.27 ^b^	2.66 ^a^	4.66 ^a^
Trial 3	5.55 ^b^	6.93 ^a^	1.79 ^b^
Silkie bantam*trial 1	100.44 ^a^	3.11 ^a^	4.63 ^abc^
Silkie bantam*trial 2	11.11 ^bc^	1.33 ^b^	3.13 ^bc^
Silkie bantam*trial 3	8.00 ^bc^	2.00 ^b^	1.25 ^c^
Pekin bantam*trial 1	32.42 ^bc^	1.83 ^b^	3.00 ^c^
Pekin bantam*trial 2	8.42 ^c^	3.33 ^b^	2.50 ^c^
Pekin bantam*trial 3	2.17 ^c^	0.75 ^b^	1.25 ^c^
Ko-Shamo*trial 1	70.78 ^ab^	9.00 ^ab^	12.67 ^a^
Ko-Shamo*trial 2	3.33 ^c^	4.55 ^ab^	11.11 ^ab^
Ko-Shamo*trial 3	3.44 ^c^	23.67 ^a^	2.67 ^c^
Chabo daruma*trial 1	25.30 ^bc^	2.40 ^b^	4.00 ^bc^
Chabo daruma*trial 2	50.20 ^abc^	1.40 ^b^	1.90 ^c^
Chabo daruma*trial 3	8.60 ^bc^	1.30 ^b^	2.00 ^c^

**Table 7:** Results of Dunn’s test with Benjamini–Hochberg correction (FDR) for comparison of results of the predisposition test of the studied chickens for chicken-assisted therapy. ** denotes statistically significant results (*p* < 0.05), * denotes statistical tendencies (*p* ≤ 0.05 < 0.10).

Compared Pair of Breeds	*p*	*p* (FDR/B-H)
Pekin bantam × Ko-Shamo	0.015 *	0.066 **
Pekin bantam × Silkie bantam	0.022 *	0.066 **
Pekin bantam × Chabo daruma	0.042 *	0.084
Ko-Shamo × Chabo daruma	0.655	0.886
Silkie bantam × Chabo daruma	0.764	0.886
Silkie bantam × Ko-Shamo	0.764	0.886

## Data Availability

The raw data supporting the conclusions of this article will be made available by the authors on request.

## Supplementary Materials

The following supporting information can be downloaded at https://www.mdpi.com/article/10.3390/ani16101429/s1. Table S1. Partial association values of the models (breed*reaction and trial*reaction) for reaction after stimulus action in subsequent tests. Table S2. Table of standardized residual values for log-linear analysis of tables and counts in breed*reaction models. The result is statistically significant if the value is <−1.96 or >1.96. Significant values are marked with **. while trends (values < −1.0 or >1.0) are marked with *. Table S3. Table of standardized residual values for log−linear analysis of tables and counts in trial*reaction models for tests SAS (Sudden Acoustic Stimuli) and UHB (Unexpected Human Behavior). The result is statistically significant if the value is <−1.96 or >1.96. Significant values are marked with **. while trends (values < −1.0 or >1.0) are marked with *.

## Abbreviations

The following abbreviations are used in this manuscript:

AAT	Animal-Assisted Therapy
CAT	Chicken-Assisted Therapy
AP	Approach People
HP	Human Presence
SAS	Sudden Acoustic Stimuli
TI	Tonic Immobility
TR	Touch Response
UHB	Unexpected Human Behavior
