# Supplementary material for: Assessment of Behavioral Predispositions of Selected Chicken Breeds for Use in Animal-Assisted Therapy: A Pilot Study
Source: Animals (Basel). 2026 May 7;16(10):1429. doi: 10.3390/ani16101429 (PMC13203667; doi:10.3390/ani16101429)
Supplement: Supplementary file 1 [file animals-16-01429-s001.zip › animals-4291176-supplementary.pdf]

Table S1. Partial association values of the models (breed*reaction and trial*reaction) for reaction after stimulus action in subsequent tests.

**Test**	*p*	χ^2	*df*
Dropping bowls Breed*reaction	0.001	27.371	9
Dropping bowls Trial*reaction	0.031	13.885	6
Clapping hands Breed*reaction	0.003	25.240	9
Clapping hands Trial*reaction	0.151	9.435	6
Loud cough Breed*reaction	0.000	35.607	9
Loud cough Trial*reaction	0.003	19.944	6
Jump Breed*reaction	0.006	23.187	9
Jump Trial*reaction	0.000	27.429	6
Throw marker Breed*reaction	0.236	11.606	9
Throw marker Trial*reaction	0.647	4.221	6
Arm swing Breed * reaction	0.381	9.633	9
Arm swing Trial*reaction	0.396	6.249	6

Table S2. Table of standardized residual values for log-linear analysis of tables and counts in breed*reaction models. The result is statistically significant if the value is < −1.96 or > 1.96. Significant values are marked with **. while trends (values < −1.0 or > 1.0) are marked with *.

Test and model	Trial	Silkie bantam	Pekin bantam	Ko-Shamo	Chabo daruma
Dropping bowls Breed*reaction	1	Freeze = 0.776<br>Looking = −1.443*<br>Move = −0.276<br>None = 0.000	Freeze = −0.765<br>Looking = 0.342<br>Move = 0.353<br>None = 0.435	Freeze = 0.000<br>Looking = 0.217<br>Move = 0.693<br>None = −0.701	Freeze = −0.342<br>Looking = 0.878<br>Move = −0.796<br>None = 0.365
	2	Freeze = −0.406<br>Looking = 0.677<br>Move = 0.746<br>None = −0.500	Freeze = 1.443*<br>Looking = 0.317<br>Move = −0.848<br>None = −0.666	Freeze = −1.060*<br>Looking = 0.257<br>Move = −0.725<br>None = 1.178*	Freeze = 0.056<br>Looking = −1.075*<br>Move = 1.001*<br>None = −0.129
	3	Freeze = −0.877<br>Looking = 0.864<br>Move = −0.637<br>None = 0.500	Freeze = −0.608<br>Looking = −0.591<br>Move = 0.693<br>None = 0.359	Freeze = 1.500*<br>Looking = −0.420<br>Move = 0.285<br>None = −0.682	Freeze = 0.560<br>Looking = 0.009<br>Move = −0.520<br>None = −0.129
Clapping hands Breed*reaction	1	Freeze = −1.171*<br>Looking = 1.157*<br>Move = 0.048<br>None = 0.288	Freeze = −0.07<br>Looking = 1.156*<br>Move = −0.1496<br>None = −0.749	Freeze = −0.846<br>Looking = −0.353<br>Move = 0.084<br>None = 1.010*	Freeze = 1.428*<br>Looking = −1.714*<br>Move = −0.149<br>None = −0.179
	2	Freeze = 1.172<br>Looking = −0.682<br>Move = −0.114<br>None = 0.138	Freeze = 0.109<br>Looking = 0.255<br>Move = −0.245<br>None = −0.060	Freeze = −0.314<br>Looking = −0.499<br>Move = 0.251<br>None = −0.485	Freeze = −0.866<br>Looking = 0.771<br>Move = −0.245<br>None = 0.450
	3	Freeze = 0.396<br>Looking = −0.267<br>Move = 0.137<br>None = −0.416	Freeze = 0.000<br>Looking = −1.181*<br>Move = 0.770<br>None = 0.780	Freeze = 1.234*<br>Looking = 0.771<br>Move = −0.661<br>None = −0.485	Freeze = −0.942<br>Looking = 0.630<br>Move = 0.770<br>None = −0.278
Loud cough Breed*reaction	1	Freeze = 0.290<br>Looking = −0.365<br>Move = 0.262<br>None = −0.751	Freeze = 2.012**<br>Looking = −0.226<br>Move = −0.424<br>None = −0.306	Freeze = −1.692*<br>Looking = −0.226<br>Move = 0.897<br>None =0.947	Freeze = −0.698<br>Looking = −0.470<br>Move = −0.735<br>None = 0.056

	2	Freeze = −0.336<br>Looking = 0.510<br>Move = 0.282<br>None = −0.231	Freeze = −0.959<br>Looking = 0.088<br>Move = 0.282<br>None = 0.186	Freeze = 0.562<br>Looking = −0.328<br>Move = 0.261<br>None = −0.231	Freeze = 0.683<br>Looking = −0.519<br>Move = −0.567<br>None = 0.560
	3	Freeze = 0.039<br>Looking = −0.288<br>Move = −0.633<br>None = 0.691	Freeze = −1.427*<br>Looking = 0.035<br>Move = 0.282<br>None = 0.132	Freeze = 1.504*<br>Looking = 0.558<br>Move = −1.458*<br>None = −0.592	Freeze = 0.060<br>Looking = −0.608<br>Move = 1.548*<br>None = −0.342
Jump<br>Breed*reaction	1	Freeze = −0.101<br>Looking = −0.710<br>Move = −0.237<br>None = 0.272	Freeze = 0.264<br>Looking = 0.336<br>Move = 0.166<br>None =0.018	Freeze = 0.132<br>Looking = 0.018<br>Move = 0.823<br>None = −0.310	Freeze = −0.470<br>Looking = 0.212<br>Move = −0.591<br>None = 0.223
	2	Freeze = 0.098<br>Looking = 0.246<br>Move = −0.337<br>None = 0.032	Freeze = 1.075*<br>Looking = −0.204<br>Move = −0.098<br>None = −1.090*	Freeze = −0.406<br>Looking = −0.220<br>Move = −0.401<br>None = 1.091*	Freeze = −1.189*<br>Looking = 0.119<br>Move = 0.869<br>None = −0.188
	3	Freeze = −0.024<br>Looking = 1.131*<br>Move = 0.778<br>None = −0.220	Freeze = −0.920<br>Looking = −0.410<br>Move = −0.100<br>None = 0.899	Freeze = 0.237<br>Looking = 0.272<br>Move = −0.607<br>None = −0.693	Freeze = 1.1032*<br>Looking = −0.612<br>Move = −0.335<br>None = 0.000
Throw marker<br>Breed*reaction	1	Freeze = 0.456<br>Looking = −0.046<br>Move = −0.376<br>Vocalization = −0.204	Freeze = 0.449<br>Looking = −0.188<br>Move = 0.888<br>Vocalization = −0.973	Freeze = −0.158<br>Looking = 0.359<br>Move = −0.545<br>Vocalization = 1.134*	Freeze = −0.793<br>Looking = −0.038<br>Move = 0.058<br>Vocalization = 0.400
	2	Freeze = 0.353<br>Looking = −0.559<br>Move = −0.136<br>Vocalization = 1.010*	Freeze = −0.158<br>Looking = 0.899<br>Move = −0.463<br>Vocalization = −0.588	Freeze = 0.204<br>Looking = −0.254<br>Move = −0.024<br>Vocalization = 0.015	Freeze = −0.400<br>Looking = −0.265<br>Move = 0.571<br>Vocalization = −0.094
	3	Freeze = −0.816<br>Looking = 0.631<br>Move = 0.494<br>Vocalization = −0.816	Freeze = −0.365<br>Looking = −0.705<br>Move = −0.361<br>Vocalization = 1.203*	Freeze = 0.000<br>Looking = −0.158<br>Move = 0.538<br>Vocalization = −0.770	Freeze = 1.234*<br>Looking = 0.319<br>Move = −0.641<br>Vocalization = −0.178
Arm swing<br>Breed*reaction	1	Freeze = 0.028<br>Looking = −0.452<br>Move = 0.344<br>None = 0.021	Freeze = 1.129*<br>Looking = 0.226<br>Move = −1.030*<br>None = 0.021	Freeze = −0.880<br>Looking = 0.000<br>Move = 0.933<br>None = −0.288	Freeze = −0.451<br>Looking = 0.226<br>Move = −0.010<br>None = 0.343

2	Freeze = 0.396<br>Looking = 0.015<br>Move = −0.266<br>None = −0.460	Freeze = 0.303<br>Looking = −0.620<br>Move = 0.068<br>None = −0.460	Freeze = 0.534<br>Looking = −0.094<br>Move = −0.191<br>None = 0.367	Freeze = −1.191*<br>Looking = 0.650<br>Move = 0.372<br>None = 0.358
3	Freeze = −0.396<br>Looking = 0.471<br>Move = −0.037<br>None = 0.286	Freeze = −1.272*<br>Looking = 0.471<br>Move = 0.984<br>None = 0.286	Freeze = 0.267<br>Looking = 0.109<br>Move = −0.737<br>None = 0.066	Freeze = 1.513<br>Looking = −0.99<br>Move = −0.433<br>None = −0.614

Table S3. Table of standardized residual values for log−linear analysis of tables and counts in trial*reaction models for tests SAS (Sudden Acoustic Stimuli) and UHB (Unexpected Human Behavior). The result is statistically significant if the value is < −1.96 or > 1.96. Significant values are marked with **. while trends (values < −1.0 or > 1.0) are marked with *.

Test and model	Breed	Trial 1	Trial 2	Trial 3
Dropping bowls Trial*reaction	Silkie bantam	Freeze = 0.776<br>Looking = −1.443*<br>Move = −0.276<br>None = 0.000	Freeze = −0.406<br>Looking = 0.677<br>Move = 0.746<br>None = −0.500	Freeze = −0.877<br>Looking = 0.864<br>Move = −0.637<br>None = 0.500
	Pekin bantam	Freeze = −0.765<br>Looking = 0.342<br>Move = 0.353<br>None = 0.435	Freeze = 1.443*<br>Looking = 0.317<br>Move = −0.849<br>None =−0.666	Freeze = −0.608<br>Looking = −0.591<br>Move = 0.693<br>None =0.359
	Ko−Shamo	Freeze = 0.000<br>Looking = 0.217<br>Move = 0.693<br>None = −0.701	Freeze = −1.060*<br>Looking = 0.257<br>Move = −0.725<br>None = 1.178*	Freeze = 1.500*<br>Looking = −0.420<br>Move = 0.285<br>None = −0.682
	Chabo daruma	Freeze = −0.342<br>Looking = 0.878<br>Move = −0.796<br>None = 0.365	Freeze = 0.056<br>Looking = −1.075*<br>Move = 1.001*<br>None = −0.129	Freeze = 0.560<br>Looking = 0.009<br>Move = −0.520<br>None =−0.129

Clapping hands Trial*reaction	Silkie bantam	Freeze = −1.171*<br>Looking = 1.157*<br>Move = 0.048<br>None = 0.288	Freeze = 1.172*<br>Looking = −0.682<br>Move = −0.114<br>None = 0.138	Freeze = 0.396<br>Looking = −0.267<br>Move = 0.137<br>None = −0.416
	Pekin bantam	Freeze = −0.077<br>Looking = 1.156*<br>Move = −0.149<br>None = −0.749	Freeze = 0.109<br>Looking = 0.255<br>Move = −0.245<br>None = −0.060	Freeze = 0.000<br>Looking = −1.181*<br>Move = 0.770<br>None = 0.780
	Ko–Shamo	Freeze = −0.846<br>Looking = −0.353<br>Move = 0.084<br>None = 1.010*	Freeze = −0.314<br>Looking = −0.499<br>Move = 0.251<br>None = −0.485	Freeze = 1.234*<br>Looking = 0.771<br>Move = −0.661<br>None = −0.485
	Chabo daruma	Freeze = 1.428<br>Looking = −1.713*<br>Move = −0.1496<br>None = −0.179	Freeze = −0.866<br>Looking = 0.771<br>Move = −0.245<br>None = 0.450	Freeze = −0.942<br>Looking = 0.630<br>Move = 0.770<br>None = −0.278
Loud cough Trial*reaction	Silkie bantam	Freeze = 0.290<br>Looking = −0.365<br>Move = 0.2627<br>None = −0.751	Freeze = −0.336<br>Looking = 0.510<br>Move = 0.282<br>None =−0.231	Freeze = 0.039<br>Looking = −0.288<br>Move = −0.633<br>None = 0.691
	Pekin bantam	Freeze = 2.012**<br>Looking = −0.226<br>Move = −0.4244<br>None = −0.306	Freeze = −0.959<br>Looking = 0.088<br>Move = 0.282<br>None = 0.186	Freeze = −1.427<br>Looking = 0.035<br>Move = 0.282<br>None = 0.132
	Ko–Shamo	Freeze = −1.692*<br>Looking = −0.470<br>Move = 0.897<br>None = 0.947	Freeze = 0.562<br>Looking = −0.328<br>Move = 0.261<br>None = −0.231	Freeze = 1.504*<br>Looking = 0.558<br>Move = −1.458*<br>None = −0.592
	Chabo daruma	Freeze = −0.698<br>Looking = 2.083**<br>Move = −0.735<br>None = 0.056	Freeze = 0.683<br>Looking = −0.519<br>Move = −0.567<br>None = 0.560	Freeze = 0.060<br>Looking = −0.608<br>Move = 1.548*<br>None = −0.342
Jump Trial*reaction	Silkie bantam	Freeze = −0.101<br>Looking = −0.710<br>Move = −0.237<br>None = 0.272	Freeze = 0.098<br>Looking = 0.246<br>Move = −0.337<br>None = 0.032	Freeze = −0.024<br>Looking = 1.131*<br>Move = 0.778<br>None = −0.220

	Pekin bantam	Freeze = 0.264<br>Looking = 0.336<br>Move = 0.166<br>None = 0.018	Freeze = 1.075*<br>Looking = −0.204<br>Move = −0.098<br>None = −1.090*	Freeze = −0.920<br>Looking = −0.410<br>Move = −0.100<br>None = 0.899
	Ko–Shamo	Freeze = 0.132<br>Looking = 0.018<br>Move = 0.823<br>None = −0.310	Freeze = −0.406<br>Looking = −0.220<br>Move = −0.401<br>None = 1.091*	Freeze = 0.237<br>Looking = 0.272<br>Move = −0.607<br>None = −0.693
	Chabo daruma	Freeze = −0.470<br>Looking = 0.212<br>Move = −0.591<br>None = 0.223	Freeze = −1.189*<br>Looking = 0.119<br>Move = 0.869<br>None = −0.188	Freeze = 1.103*<br>Looking = −0.612<br>Move = −0.335<br>None = 0.000
Throw marker Trial*reaction	Silkie bantam	Freeze = 0.456<br>Looking = −0.046<br>Move = −0.376<br>Vocalization = −0.204	Freeze = 0.353<br>Looking = −0.559<br>Move = −0.136<br>Vocalization = 1.010*	Freeze = −0.816<br>Looking = 0.631<br>Move = 0.494<br>Vocalization = −0.816
	Pekin bantam	Freeze = 0.449<br>Looking = −0.188<br>Move = 0.888<br>Vocalization = −0.973	Freeze = −0.158<br>Looking = 0.899<br>Move = −0.463<br>Vocalization = −0.588	Freeze = −0.365<br>Looking = −0.705<br>Move = −0.361<br>Vocalization = 1.203*
	Ko–Shamo	Freeze = −0.158<br>Looking = 0.359<br>Move = −0.545<br>Vocalization = 1.134*	Freeze = 0.204<br>Looking = −0.254<br>Move = −0.024<br>Vocalization = 0.015	Freeze = 0.000<br>Looking = −0.158<br>Move = 0.538<br>Vocalization = −0.770
	Chabo daruma	Freeze = −0.793<br>Looking = −0.038<br>Move = 0.058<br>Vocalization = 0.400	Freeze = −0.400<br>Looking = −0.265<br>Move = 0.571<br>Vocalization = −0.094	Freeze = 1.234*<br>Looking = 0.319<br>Move = −0.641<br>Vocalization = −0.178
Arm swing Trial*reaction	Silkie bantam	Freeze = 0.028<br>Looking = −0.452<br>Move = 0.344<br>None = 0.021	Freeze = 0.396<br>Looking = 0.015<br>Move = −0.266<br>None = −0.460	Freeze = −0.396<br>Looking = 0.471<br>Move = −0.037<br>None = 0.286

Pekin bantam	Freeze = 1.129*<br>Looking = 0.226<br>Move = −1.030*<br>None = 0.021	Freeze = 0.303<br>Looking = −0.620<br>Move = 0.068<br>None = −0.460	Freeze = −1.272*<br>Looking = 0.471<br>Move = 0.984<br>None = 0.286
Ko–Shamo	Freeze = −0.880<br>Looking = 0.000<br>Move = 0.933<br>None = −0.288	Freeze = 0.534<br>Looking = −0.094<br>Move = −0.191<br>None = 0.367	Freeze = 0.267<br>Looking = 0.109<br>Move = −0.737<br>None = 0.066
Chabo daruma	Freeze = −0.451<br>Looking = 0.226<br>Move = −0.010<br>None = 0.343	Freeze = −1.191*<br>Looking = 0.650<br>Move = 0.372<br>None = 0.358	Freeze = 1.513*<br>Looking = −0.995<br>Move = −0.433<br>None = −0.614
